# Genome-wide association analyses of child genotype effects and parent-of-origin effects in specific language impairment

**DOI:** 10.1111/gbb.12127

**Published:** 2014-03-24

**Authors:** R Nudel, N H Simpson, G Baird, A O’Hare, G Conti-Ramsden, P F Bolton, E R Hennessy, S M Ring, G Davey Smith, C Francks, S Paracchini, A P Monaco, S E Fisher, D F Newbury

**Affiliations:** † Wellcome Trust Centre for Human Genetics, University of OxfordOxford, UK; ‡ Newcomen Centre, the Evelina Children’s HospitalLondon, UK; § Department of Reproductive and Developmental Sciences, University of EdinburghEdinburgh, UK; ¶ School of Psychological Sciences, University of ManchesterManchester, UK; ** Departments of Child & Adolescent Psychiatry & Social Genetic & Developmental Psychiatry Centre, Institute of Psychiatry, King’s College LondonLondon, UK; †† University Child Health and DMDE, University of AberdeenAberdeen, UK; ‡‡ School of Social and Community Medicine, University of BristolBristol, UK; §§ MRC Integrative Epidemiology Unit, University of BristolBristol, UK; ¶¶ Max Planck Institute for PsycholinguisticsNijmegen, the Netherlands; *** Donders Institute for Brain, Cognition and Behaviour, Radboud UniversityNijmegen, the Netherlands; ††† School of Medicine, University of St AndrewsSt Andrews, UK; ‡‡‡ Tufts UniversityMedford, MA, USA

**Keywords:** ALSPAC, GWAS, imprinting, neurodevelopmental disorder, specific language impairment

## Abstract

**Specific language impairment (SLI) is a neurodevelopmental disorder that affects linguistic abilities when development is otherwise normal. We report the results of a genome-wide association study of SLI which included parent-of-origin effects and child genotype effects and used 278 families of language-impaired children. The child genotype effects analysis did not identify significant associations. We found genome-wide significant paternal parent-of-origin effects on chromosome 14q12 (*P* = 3.74 × 10^−8^) and suggestive maternal parent-of-origin effects on chromosome 5p13 (*P* = 1.16 × 10^−7^). A subsequent targeted association of six single-nucleotide-polymorphisms (SNPs) on chromosome 5 in 313 language-impaired individuals and their mothers from the ALSPAC cohort replicated the maternal effects, albeit in the opposite direction (*P* = 0.001); as fathers’ genotypes were not available in the ALSPAC study, the replication analysis did not include paternal parent-of-origin effects. The paternally-associated SNP on chromosome 14 yields a non-synonymous coding change within the *NOP9* gene. This gene encodes an RNA-binding protein that has been reported to be significantly dysregulated in individuals with schizophrenia. The region of maternal association on chromosome 5 falls between the *PTGER4* and *DAB2* genes, in a region previously implicated in autism and ADHD. The top SNP in this association locus is a potential expression QTL of *ARHGEF19* (also called *WGEF*) on chromosome 1. Members of this protein family have been implicated in intellectual disability. In summary, this study implicates parent-of-origin effects in language impairment, and adds an interesting new dimension to the emerging picture of shared genetic etiology across various neurodevelopmental disorders**.

Specific language impairment (SLI) is a complex and heterogeneous neurodevelopmental disorder diagnosed when the child has difficulties with language development despite otherwise showing normal development (Bishop 2006). Specific language impairment affects approximately 7% of preschool children (Tomblin *et al.* 1997). Familial aggregation and twin studies of SLI indicate a strong genetic component: across several studies, siblings of probands had a 30.3% mean rate of affectedness (higher than in the general population), and a meta-analysis of twin studies showed overall concordances of 83.6% for monozygotic twins and 50.2% for dizygotic twins (Stromswold 1998, 2001). A study of SLI twins which examined quantitative language scores found significant heritability estimates close to 1 for several language scores which account for both expressive and receptive language skills, although these effects were not present when controlling for IQ (Bishop *et al.* 1995). Further studies also obtained significant heritability estimates, as reviewed by Stromswold (2001). In terms of the prevalence of SLI, males are more frequently affected compared to females (Stromswold 1998), and relatives of males seem to be more frequently affected compared to relatives of females (Conti-Ramsden *et al.* 2007). However, a twin study that examined same-sex monozygotic twins and opposite-sex dizygotic twins concluded that there were no sex-specific genetic effects on language impairment that can explain the differences in the prevalence between males and females (Viding *et al.* 2004). Some studies suggest that the sex-bias simply represents a selection bias of males (Stromswold 1998).

Linkage and targeted association studies of SLI have identified several chromosomal regions and genes as candidates for involvement in susceptibility. These include: chromosome 7 (OMIM#602514) (Villanueva *et al.* 2011), chromosome 13 (OMIM#607134) (Bartlett *et al.* 2004, 2002), and chromosomes 16 (OMIM#606711) and 19 (OMIM#606712) (Falcaro *et al.* 2008; Monaco 2007; The SLI Consortium 2002, 2004), *CNTNAP2* (OMIM#604569), *CMIP* (OMIM#610112) and *ATP2C2* (OMIM#613082) (Newbury *et al.* 2009; Vernes *et al.* 2008)

Previous genome-wide analyses of SLI utilized various linkage methods: parametric or non-parametric, and quantitative or categorical. Certain assumptions may affect the choice of linkage method, including the definition of language impairment itself, which could lead to differences in the loci identified. Linkage may be more efficient than association when different mutations in the same gene contribute to the disorder across families, rather than a specific set of alleles. Association may be more powerful than linkage in detecting variants of small effect sizes, which all contribute to the risk, as expected in complex disorders (Burmeister *et al.* 2008; Risch & Merikangas 1996; Risch 2000).

Genome-wide association studies of SLI, *per se*, have yet to be reported but groups have investigated genome-wide effects in related traits. Meaburn *et al.* (2008) applied a pooled genotyping method across cases and controls with low or high reading abilities from a population cohort, but did not identify any significant associations. In their dyslexia study, Field *et al.* (2013) reported suggestive association with a region downstream of *FGF18*. Luciano *et al.* (2013) investigated quantitative reading and language measures across two population samples and found suggestive association with variants in *ABCC13*. Eicher *et al.* (2013) used individuals with low-language and/or reading performance and control individuals in a genome-wide association screen. They reported suggestive association between co-morbid language and reading problems and single-nucleotide-polymorphisms (SNPs) in *ZNF385D* and *COL4A2.* Their screen of individuals affected only by oral language deficits highlighted SNPs in *NDST4*.

When parent-of-origin effects are present, the expression of an allele depends on which parent it was inherited from. Imprinting is an epigenetic phenomenon that results in a parent-of-origin effect. Imprinted genes expressed in the brain are involved in various aspects of neurodevelopmental processes, including: neuronal differentiation, growth and gene regulation, and may even affect the behavior in animal models during their adult lives (Wilkinson *et al.* 2007). There is some evidence to suggest that epigenetic modifications, including imprinting, may play a role in the etiology of autism (Schanen 2006). Other neurodevelopmental disorders, such as Prader-Willi syndrome and Angelman syndrome, both of which include language deficits, involve imprinted genomic regions, as reviewed by Chamberlain and Lalande (2010). Allele-biased expression, which may result from imprinting, has been reported for genes implicated in autism and schizophrenia (Lin *et al.* 2012). It has recently been shown that the expression of non-imprinted genes in the mouse may still display parent-of-origin effects through interaction with imprinted loci, and that this phenomenon is a contributing factor in the genetic architecture of complex traits (Mott *et al.* 2014).

We applied the EMIM tool, which uses family subsets, to test for parent-of-origin effects and child genotype effects. When parent-of-origin effects are present, the EMIM methodology allows for a more accurate model and, consequently, improved detection of associations, compared to that of traditional case–control association methods (Ainsworth *et al.* 2011).

## Materials and methods

### Participants

The final (post-quality control) sample included 278 nuclear families (which included 297 affected children) from the SLI Consortium (SLIC) cohort, including samples from five centers across the UK (The Newcomen Centre at Guy’s Hospital, London (now called Evelina Children’s Hospital); the Cambridge Language and Speech Project (CLASP); the Child Life and Health Department at the University of Edinburgh; the Department of Child Health at the University of Aberdeen; and the Manchester Language Study), as described in previous SLI Consortium studies (Falcaro *et al.* 2008; Monaco 2007; Newbury *et al.* 2009; The SLI Consortium 2002, 2004). The 278 families included 49 families from the Guy’s Hospital, London cohort which had not been included in previous SLI Consortium studies. All participants had a ‘white British’ background (a principal component analysis was carried out, and samples were excluded based on divergent ancestry, see section on the genotype arrays below). The proband from each family was defined as a case. Any sibling who had an expressive language score or a receptive language score, as obtained with the revised version of the Clinical Evaluation of Language Fundamentals (CELF) (Semel *et al.* 1992), of 1.5 SD or more below the general population mean for their age was also defined as a case. All cases had a WISC Perceptual Organization Index (a composite score of the non-verbal subtests Picture Completion, Picture Arrangement, Block Design and Object Assembly) of >77.5 (1.5 SD below that expected for their age) and did not have a diagnosis of autism or hearing impairment. Siblings who had both expressive and receptive language scores above the mean were defined as controls. Note that controls did not contribute to the analyses *per se* but were used in the derivation of expected minor allele frequencies. We did not exclude children on the basis of a diagnosis of ADHD or dyslexia alone, given the high degree of co-occurrence of SLI and ADHD or dyslexia. However, for some of our SLIC samples, data were available for the presence of hyperactivity, coordination and reading problems. From this, we estimate that approximately one third of our SLIC samples showed some evidence of ADHD or developmental coordination disorder, and that approximately one half of our probands had reading problems.

Ethical agreement for the SLIC study was given by local ethics committees, and all subjects provided informed consent.

### Replication cohort

The replication cohort for the maternal parent-of-origin effects analysis comprised 313 language-impaired children and their mothers from the ALSPAC cohort (Boyd *et al.* 2013; Fraser *et al.* 2013). We were looking for a replication cohort consisting of children who have been administered language tests and their parents who have additionally been genotyped; the ALSPAC cohort suited that purpose. However, fathers were not genotyped, and therefore paternal parent-of-origin effects were not investigated in this replication cohort. We aimed to select a subset of the ALSPAC cohort that best resembled the SLIC cohort. Preliminary filtering of the cohort therefore involved the exclusion of children who were not of a self-described ‘white’ ethnicity, had hearing problems, had a PIQ score of below 80, or were diagnosed with autism or pervasive developmental disorder. The ALSPAC cohort was not administered the CELF, so we instead selected cases based upon alternative language-related phenotypes: the speech and syntax subscales, from the Children’s Communication Checklist (CCC) (Bishop 1998) to represent expressive language skills, and the comprehension subtest of the Wechsler Objective Language Dimensions (WOLD) (Rust 1996) to represent receptive language skills. Cases were defined as having an expressive (on both speech and syntax subscales) or receptive language score (the WOLD comprehension subtest) at least 1.5 SD below the mean of the entire ALSPAC cohort (after the preliminary filtering). Additionally, all cases had a pragmatic composite score from the CCC of 132 or above, as this cutoff was deemed efficient in discriminating children with SLI from children with pragmatic language impairment, and, potentially, children who might have autism (Bishop & Norbury 2002; Bishop 1998). With regards to the ALSPAC cohort, please note that the study website contains details of all the data that are available through a fully searchable data dictionary (http://www.bris.ac.uk/alspac/researchers/data-access/data-dictionary).

### Genotype array and quality control measures

Blood and mouth swab samples were collected and the genomic DNA was extracted by standard protocols. Genomic DNA was quantified using the Quant-iT Pico Green dsDNA Assay Kit (Invitrogen, Grand Island, NY, USA); samples with low amounts of DNA were subject to whole-genome amplification prior to genotyping. Samples were genotyped using the Illumina Human Omni-Express (v12.1) array (Illumina, San Diego, CA, USA). Samples were randomized across plates, with cases and controls being spread evenly across plates, as were different sample types and samples of different origins, while family units were retained with plates. Forty seven samples were duplicated across plates (concordance rate = 0.98968), and checks for inter-plate variances were performed. Quality control measures, as described in Anderson *et al.* (2010), were performed in two steps: prior to performing the association analyses, SNPs and samples were filtered based on several quality control measures: SNPs and samples with a genotype success rate below 95% and/or heterozygosity rates ±2 SD from the mean were removed, as were all SNPs with a minor allele frequency of less than 1%. Single-nucleotide-polymorphisms with a Gentrain score below 0.5 were removed (Gentrain is a clustering algorithm which produces a score based on the shapes of the genotype clusters of a given SNP and their distances from each other). Single-nucleotide-polymorphisms and samples with an error rate of 1% or higher, as estimated by bad inheritances, were removed. Inheritance data within families were used to exclude SNPs and samples with an error rate of above 1%. Control data (Hapmap release #3) were employed through a principal component analysis to exclude individuals with divergent ancestry, and samples with gross chromosome rearrangements or discordant sex information were removed. In total, 74 individuals from 40 families were removed following the principal component analysis or due to having chromosome rearrangements, extreme heterozygosity rates or discordant sex information. We used PEDSTATS (Wigginton & Abecasis 2005) to make sure no Mendelian errors were present in the final pedigree file. PLINK (Purcell *et al.* 2007) was used to exclude SNPs based on Hardy-Weinberg equilibrium (HWE) *P*-values, with a threshold of 0.001. Genotypes were compared with existing SNP genotype data for 700 individuals and 734 SNPs, with an error rate of 0.00967. The total number of SNPs used in the association analyses was 614 937. Following the association analyses, we reexamined the HWE *P*-values of all SNPs with an association *P* < 10^−4^ using PEDSTATS, which selects unrelated samples in a different way from that used by PLINK, resulting in a more powerful test due to increased sample sizes. We removed SNPs that had HWE *P* < 0.001 if they were not supported by adjacent SNPs (i.e. SNPs with very low P and HWE *P*-values for which adjacent SNPs did not show any association were removed), and the results were plotted. SNPs that formed association peaks, i.e. a cluster of SNPs in which one SNP has a significant *P*-value and adjacent SNPs on both sides have slightly higher *P*-values, were then checked manually for good clustering with Genome Studio.

In the replication analysis, six SNPs that showed association in the SLIC cohort were investigated in the ALSPAC cohort. The ALSPAC samples were genotyped using the Illumina human 660 W-quad array (Illumina, San Diego, CA, USA) (mothers) or the Illumina Human Hap 550-quad array (Illumina, San Diego, CA, USA) (children). ALSPAC quality control measures included the removal of SNPs with more than 5% missing genotype rate, or minor allele frequency of less than 1%. Single-nucleotide-polymorphisms with HWE *P*-values of less than 10^−6^ (mothers) or 5 × 10^−7^ (children) were removed. Samples with a missing genotype rate of more than 5% (mothers) or 3% (children) were removed. Other exclusion criteria included incorrect gender assignments and extreme heterozygosity rates. We found no Mendelian errors for any mother–child duo with the SNPs used in our analysis.

### Statistical analyses

We used EMIM (Ainsworth *et al.* 2011; Howey & Cordell 2012) to perform the association analyses. EMIM estimates the effects of one or more parameters on the increase in the risk of having the disorder. In our analyses of the SLIC cohort, one parameter was estimated in each analysis. These were:

1. *R*_1_: the factor by which the risk is multiplied when the child has a single copy of the risk allele (Ainsworth *et al.* 2011). In this analysis, the increase in risk when the child has two risk alleles is assumed to be *R*_1_^2^ (this is sometimes referred to as a child trend analysis in the EMIM documentation, and is the test which is the most similar to a case–control analysis).
2. *I*_p_: the factor by which the risk is multiplied when the child receives a risk allele from the father (Ainsworth *et al.* 2011; Weinberg *et al.* 1998).
3. *I*_m_: the factor by which the risk is multiplied when the child receives a risk allele from the mother (Ainsworth *et al.* 2011; Weinberg *et al.* 1998).

Simulations of similar models which used the above parameters indicated moderate to high power: with 100 case-parents trios, child genotype risk factors of 2 for having one risk allele and 3 for having two risk alleles, and a risk allele frequency of 0.1–0.3, the power was ∼68%, and the type I error rate was 0.058 (Weinberg *et al.* 1998). Note that our child trend analysis estimated only one risk parameter. When examining parent-of-origin effects with the same sample and a parent-of-origin risk factor of 2.5, the power was higher than 92%, and type I error rate was consistent with the nominal 0.05 (Weinberg *et al.* 1998). The model employed in our study allowed for the use of additional types of family subsets, which would be expected to increase the power. In addition, our sample included more than 100 case-parents trios on average per SNP, and the parent-of-origin risk factors were estimated to be higher than 2.5 for our top associations.

By default, EMIM treats minor alleles as risk alleles, but this does not mean that the minor allele necessarily increases the risk, and the estimated parameters may have values below 1 if the other allele increases the risk. EMIM estimates the likelihoods of two models, one in which there is no increase in risk resulting from the parameter operating, the null model, and another in which there is an increase in risk (given the parameter being estimated), the alternative model. The baseline risk which the parameters modify is defined as the probability of disease in a child who does not carry the risk allele. To calculate *P*-values, one may use the value of twice the difference between the maximized log-likelihoods of the two models as a *χ*^2^ statistic, with the number of degrees of freedom being the same as the number of parameters tested. EMIM assumes that individuals with missing/unknown affection statuses are controls, which holds true only for rare disorders, and, therefore, control subsets were not used in our analyses.

In the ALSPAC replication sample, in which paternal genotypes were not available, we performed a maternal parent-of-origin analysis only.

### Family subsets

Individuals were grouped into case duos and trios with their parents using PREMIM (Howey & Cordell 2012), with the −a option (which includes an estimation of the minor allele frequencies in the sample). Control subsets were not used in our analyses, but they contributed to the estimation of minor allele frequencies. The case and/or parent/s subsets are independent from each other and are constructed per SNP, i.e. if a case and their two parents were used to construct a trio, then they would not be used in any other subset for that SNP. The program built the subsets using our cohort in the following order of preference: case-parents trio, case-mother duo, case-father duo, case, parents of a case, mother of a case, and father of a case. The average numbers of these subsets per SNP in the SLIC sample were: 153 case-parents trios, 54 case-mother duos, 12 case-father duos and 18 cases. In the ALSPAC replication cohort, there were 193 case-mother duos, 75 cases, and 45 mothers of cases on average per SNP. Minor allele frequencies were estimated using all individuals present after the preliminary filtering and prior to the selection of cases.

Manhattan and QQ plots were generated in R with a script written by Stephen Turner and Daniel Capurso (https://raw.github.com/stephenturner/qqman/master/qqman.r). Plots of regions surrounding association peaks were generated with LocusZoom (Pruim *et al.* 2010).

## Results

**Figure 1:**
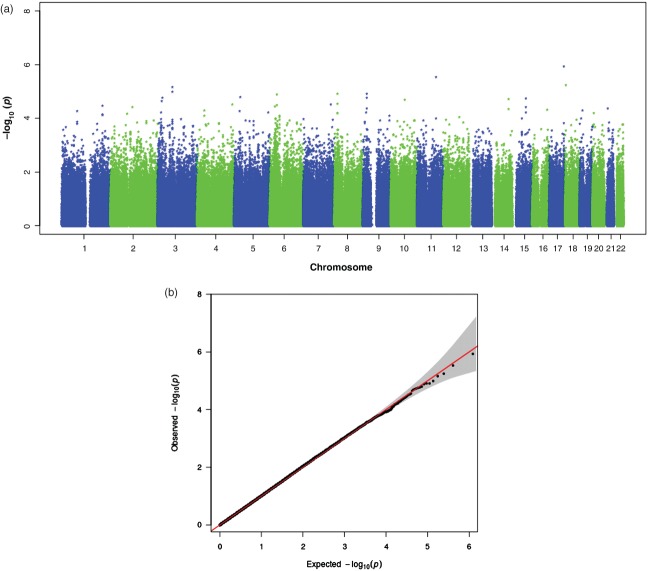
Results of the child trend association analysis. (a) Manhattan plot for the child trend association analysis. (b) QQ plot for the child trend association analysis with 95% confidence intervals.

We found significant evidence for parent-of-origin effects in SLI. We detected significant associations on chromosome 14 with paternal parent-of-origin effects and suggestive associations on chromosome 5 with maternal parent-of-origin effects. The child trend analysis did not detect any significant or suggestive associations. The results of the child trend analysis can be found in Appendix S1 and Fig. 1.

**Figure 2:**
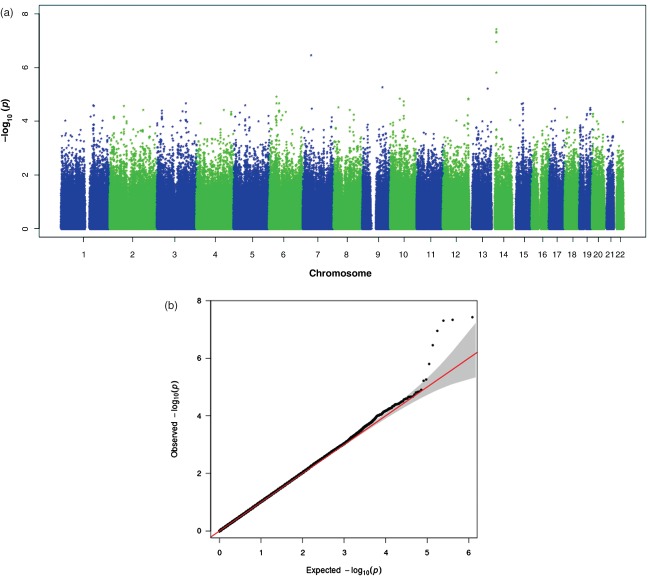
Results of the paternal parent-of-origin effects association analysis. (a) Manhattan plot for the paternal parent-of-origin effects association analysis. (b) QQ plot for the paternal parent-of-origin effects association analysis with 95% confidence intervals.

We detected paternal parent-of-origin effects on chromosomal band 14q12 (Fig. 2a). All the SNPs which formed the peak passed all quality control measures. The most significant SNP in the peak (rs4280164) reached genome-wide significance with a *P*-value of 3.74 × 10^−8^ and deviated from the ‘expected’ line on the QQ plot (Fig. 2b). The peak spans five SNPs (3.74 × 10^−8^ ≤ *P* ≤ 1.58 × 10^−6^), the left-most and right-most of which are at positions 23 839 502 and 23 856 815 (∼17 kb) (hg18), respectively. Levels of linkage disequilibrium (LD) across the associated SNPs reach *r*^2^ = 0.8.

**Figure 3:**
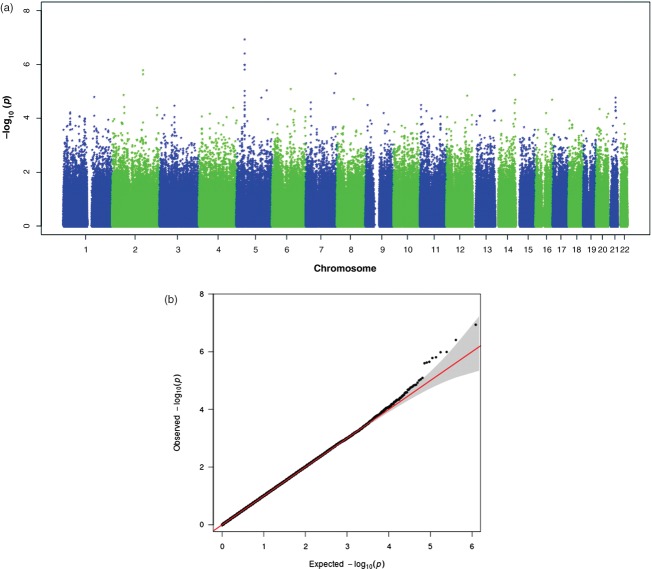
Results of the maternal parent-of-origin effects association analysis. (a) Manhattan plot for the maternal parent-of-origin effects association analysis. (b) QQ plot for the maternal parent-of-origin effects association analysis with 95% confidence intervals.

**Figure 4:**
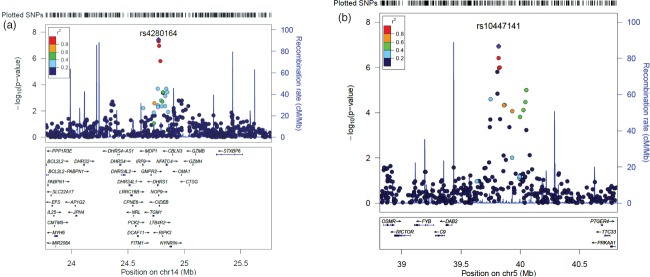
Regional association plots for top associations. (a) SNPs around the association peak on 14q12. (b) SNPs around the association peak on 5p13.

We detected maternal parent-of-origin effects on chromosomal band 5p13 (Fig. 3a). The highest SNP (rs10447141) in the peak had a *P*-value of 1.16 × 10^−7^, and, again, it deviated from the ‘expected’ line on the QQ plot (Fig. 3b). The peak of association spans ∼300 kb (39 784 227–40 086 058, hg18) and encompasses 13 SNPs. The LD levels across the associated SNPs reached *r*^2^ = 0.8. Table1 includes the *P*-values for all SNPs in the peaks on chromosomes 5 and 14, and all associations with *P* ≤ 10^−4^ are available in Appendix S1. Figure 4 includes close-up plots of the association peaks.

We performed a targeted follow-up analysis in ALSPAC with six SNPs across the SLIC maternal parent-of-origin effects association peak on chromosome 5. This analysis used children with low-language ability and their mothers from the ALSPAC cohort. In this cohort, we found a minimum *P*-value of 0.001 with SNP rs1994882, and two other SNPs had *P* ≤ 0.05. The *P*-values for the replication analyses can be found in Table1. The replicated associations in the ALSPAC cohort were in the opposite direction compared to the associations observed in the SLIC cohort.

To test whether the top association in both analyses were driven mainly by the relevant parent-of-origin effect, we adjusted the null hypothesis to assume parent-of-origin effects of the other type (i.e. when testing for paternal parent-of-origin effects, the null hypothesis will assume maternal parent-of-origin effects are present, and *vice versa*) at those loci. The *P*-values for the top SNPs were 3.87 × 10^−7^ and 1.29 × 10^−7^ in the paternal and maternal parent-of-origin analyses, respectively, suggesting that even if parental effects of the other type were present, the association is driven mainly by paternal and maternal effects, respectively.

## Discussion

In this study, we investigate parent-of-origin effects and child genotype effects in SLI by means of association analyses specifically designed to be used with family data. We detected significant and suggestive associations in the paternal and maternal parent-of-origin effects analyses, respectively. The child trend analysis did not detect significant or suggestive associations. It is possible that the child effects were not strong enough to be detected in this sample, whereas the parent-of-origin effects were.

The maternally-associated peak on 5p13 does not fall within any known gene; it lies ∼863 kbp away from *PTGER4* (Prostaglandin E Receptor 4), a member of the G-protein coupled receptor family, and ∼392 kbp away from *DAB2* (Disabled Homolog 2, Mitogen-Responsive Phosphoprotein), which is involved in cellular trafficking. Significant downregulation of *PTGER4* has been reported in brains of patients with schizophrenia (Schmitt *et al.* 2011). SNPs found between those two genes have also been associated with Crohn’s disease (Libioulle *et al.* 2007). Although intergenic, the most highly-associated SNP on 5p13 (rs10447141) is listed in the SCAN database as a potential eQTL of *ARHGEF19* (Rho Guanine Nucleotide Exchange Factor 19, also called *WGEF*) on chromosome 1 (*P* = 4 × 10^−5^ using HapMap CEU lymphoblastoid cell line samples) (Gamazon *et al.* 2010). The WGEF protein belongs to a family of activators of Rho-GTPases, which are involved in a variety of cellular signaling pathways (Van Aelst & D’Souza-Schorey 1997). It has been experimentally shown that WGEF can activate Rho-GTPases and that it induces the rearrangement of cytoskeleton (Wang *et al.* 2004). WGEF has also been shown to be involved in convergent extension, an important step in embryonic development (Tanegashima *et al.* 2008). Interestingly, a gene from the same family, *ARHGEF6* (Rac/Cdc42 guanine nucleotide exchange factor 6), has been implicated in intellectual disability (Kutsche *et al.* 2000). It is possible that the mechanism through which these genes are involved in disorders such as intellectual disability relates to their roles in dendritic development and morphology (Newey *et al.* 2005). Work performed on 3T3-L1 cells revealed that *WGEF* itself is regulated through methylation, which plays an important role in genomic imprinting (Horii *et al.* 2009). Interestingly, *WGEF* lies within the dyslexia susceptibility locus DYX8 (OMIM#608995) on chromosome 1p (Grigorenko *et al.* 2001, Rabin *et al.* 1993, Tzenova *et al.* 2004). This locus, however, was identified through standard linkage analyses.

In a targeted follow-up using six SNPs, we observe consistent association to chromosome 5 in an independent cohort of language-impaired children and their mothers: the most associated SNP has a *P*-value of 0.001. Two other SNPs also have significant p-values (*P* ≤ 0.05). Although the most associated SNP in our analysis was not included in the arrays used by ALSPAC, the six SNPs tested are in a region with high LD, and the most associated SNPs in the SLIC and ALSPAC analyses are in high LD with each other (Fig. 4b). The direction of association in the replication analysis was opposite to the one observed in SLIC, but this phenomenon is well-documented in replication studies and could be explained by considering the interactions between the causal variant and the observed variant across populations (Lin *et al.* 2007). Interestingly, the exact same effect was observed in a previous SLI association study that had used the ALSPAC cohort (Newbury *et al.* 2009).

The top SNP in the region of paternal association on chromosome 14 (rs4280164) corresponds to a missense variant in the *NOP9* gene (also known as C14orf21), yielding an S308N substitution in the encoded protein. The minor allele in our sample, which is the minor allele in the general population as well, encodes the asparagine amino acid and has a frequency of 15.8% in the HapMap CEU population. It is predicted to be ‘possibly damaging’ by PolyPhen (Adzhubei *et al.* 2010); however, in our analysis it is the major allele which increases the risk when inherited from the father. This locus was not found in the Imprinted Gene and Parent-of-origin Effect Database (Morison *et al.* 2001). The S308N substitution is found five amino acid positions away from one of the protein’s RNA-binding pumilio domains, and the serine at that position is extremely conserved among mammalian species, with a mean PhyloP score of 1.1. The NOP9 protein has RNA-binding properties according to UniProt (The UniProt Consortium 2012), and the yeast ortholog, Nop9, has been shown to bind RNA and play a role in the nuclear maturation of the ribosomal subunits (Thomson *et al.* 2007). As reviewed in (Kapeli & Yeo 2012), RNA-binding proteins have been associated with several neurological disorders. This gene in particular has been found to be significantly dysregulated in schizophrenia patients and their unaffected siblings in a study of peripheral blood gene expression (Glatt *et al.* 2011).

Both association peaks are found in regions that have been previously implicated in various neurodevelopmental disorders. The peak on chromosomal band 14q12 falls within a region implicated in a homozygosity mapping study of intellectual disability (OMIM#611095) (Abou Jamra *et al.* 2011). The maternally-associated peak on 5p13 falls within a region implicated in both autism and attention deficit hyperactivity disorder (ADHD), ADHD4 (OMIM#608906). In a genome-wide linkage study of ADHD, a region of strong linkage was found on chromosomal band 5p13 (Ogdie *et al.* 2003), with the closest marker being D5S418 (chr5:40051524–40051931, hg18), which is ∼200 kbp proximal to our top SNP (rs10447141). A subsequent fine mapping of the region confirmed the linkage (Ogdie *et al.* 2004). A pooled analysis which used the cohort from the above studies and an independent cohort found that the only common risk locus was on 5p13 (Ogdie *et al.* 2006). A genome-wide screen for autism susceptibility loci obtained the highest linkage peak (under the broad-phenotype scheme used in the study) on 5p13 at marker D5S2494 (chr5:40253968–40254209), which is ∼400 kbp proximal to our top SNP (Liu *et al.* 2001). Moreover, the majority of the contribution to the IBD sharing (the linkage method used was affected-sib pair analysis) was from the maternal side, which is in line with the maternal parent-of-origin effects in our analysis. A more recent genome-wide screen of parent-of-origin effects in autism found a maternally-linked region with a peak on 5p13.1 (Fradin *et al.* 2010), which is the location of the top SNP in our association peak. Duplications on 5p13 have also been associated with developmental delay and intellectual disability (Yan *et al.* 2009), but these do not overlap with our associations. It is not clear whether imprinting plays a role in the etiology of these disorders, or whether the region on 5p13 is imprinted regardless of the disease context, which means that only the maternal allele, in this case, will have an effect. As discussed in the *Introduction* section, even when a locus is not imprinted, it may interact with imprinted loci and thereby show parent-of-origin effects. With regard to the studies discussed above, which used linkage methods, it should be noted that linkage and association methods test for different things; while linkage methods find regions that may harbor genes involved in a disease, association methods test for the statistical correlation between genetic variants and a disease.

The association peak on chromosomal band 14q12 reached genome-wide significance in terms of the widely-used threshold of 5 × 10^−8^ as proposed by Risch and Merikangas (1996). Both associations (the top SNPs on 5p13 and 14q12) are supported by association trends in adjacent SNPs. The peak on chromosomal band 5p13 did not reach the 5 × 10^−8^ threshold; this threshold, however, was based on an adjustment for 10^6^ independent tests, while we had 614937 SNPs, many of which are in LD with each other. Furthermore, the 5 × 10^−8^ threshold may not be appropriate for some study designs (Hoggart *et al.* 2008). In particular, the methods we used are different from case–control association methods, to which this threshold is traditionally applied. Even then, some studies use different thresholds, e.g. 5 × 10^−7^ (The Wellcome Trust Case Control Consortium 2007). Given the association trends of the adjacent SNPs, the fact that the associations are more significant than expected (Fig. 3b), the good clustering for all the SNPs that form the peak and the replicated association effects, we believe that the association on chromosomal band 5p13 is not a false positive. We have performed several analyses which were all mutually exclusive in terms of the parameters that were estimated while performing them. For this reason, we present thresholds corrected for the number of SNPs, but not the number of analyses. In other words, we did not have a global null hypothesis (Krawczak 2006). We note, however, that if the *P*-values of our top associations were corrected for the total number of analyses in a Bonferroni manner, they would not remain genome-wide significant.

**Table 1:** The SNPs which form the peaks on chromosomes 5 and 14

SNP ID	Chromosome	Position (hg18)	Test	Minor allele/major allele	Increase in risk, *I*_m_/*I*_p_ relative to risk allele (SLIC)	*P* (SLIC)	*P* (ALSPAC)
rs1353835	5	39 784 227	Maternal parent-of-origin effects	C[Table-fn tf1-1]/A	2.14	2.55 × 10^−5^	Not tested
rs1994882	5	39 841 921		C[Table-fn tf1-1]/A	2.441	1.56 × 10^−6^	0.001[Table-fn tf1-2]
rs12658486	5	39 841 974		G[Table-fn tf1-1]/A	2.367	1.43 × 10^−5^	0.002[Table-fn tf1-2]
rs980306	5	39 852 592		A/G[Table-fn tf1-1]	3.008	3.91 × 10^−7^	Not tested
rs10447141	5	39 852 924		A/G[Table-fn tf1-1]	3.08	1.16 × 10^−7^	Not tested
rs17194068	5	39 857 074		G/A[Table-fn tf1-1]	2.86	1.04 × 10^−6^	0.027[Table-fn tf1-2]
rs6895329	5	39 861 497		G/A[Table-fn tf1-1]	2.926	1.02 × 10^−6^	Not tested
rs1816088	5	39 897 583		A/C[Table-fn tf1-1]	2.486	4.72 × 10^−5^	Not tested
rs618051	5	39 902 670		C/A[Table-fn tf1-1]	2.429	4.62 × 10^−5^	0.152[Table-fn tf1-2]
rs542708	5	39 968 025		A/G[Table-fn tf1-1]	2.453	8.42 × 10^−5^	Not tested
rs17218399	5	40 061 719		G/A[Table-fn tf1-1]	2.4	7.54 × 10^−5^	Not tested
rs2939378	5	40 078 002		G/A[Table-fn tf1-1]	2.29	3.43 × 10^−5^	0.561
rs1567010	5	40 086 058		G/A[Table-fn tf1-1]	2.628	9.93 × 10^−6^	0.329[Table-fn tf1-2]
rs11158632	14	23 839 502	Paternal parent-of-origin effects	C/A[Table-fn tf1-1]	3.842	4.62 × 10^−8^	Not tested
rs4280164	14	23 841 124		A/G[Table-fn tf1-1]	3.872	3.74 × 10^−8^	Not tested
rs2144494	14	23 843 226		G/A[Table-fn tf1-1]	3.832	4.94 × 10^−8^	Not tested
rs2281472	14	23 845 685		G/A[Table-fn tf1-1]	3.421	1.12 × 10^−7^	Not tested
rs3181384	14	23 856 815		A/G[Table-fn tf1-1]	3.016	1.58 × 10^−6^	Not tested

Risk allele.

Association in ALSPAC is in the opposite direction compared to SLIC.

The data presented here provide compelling and significant evidence for parent-of-origin effects in SLI on chromosomes 5 and 14, which may overlap with other disorders, namely, ADHD, autism and intellectual disability. The relationship between SLI, autism and ADHD is not yet completely understood. While it is evident that language is likely to be impaired in both autism (Kjelgaard & Tager-Flusberg 2001) and ADHD (Baird *et al.* 2000), the exact nature of the language impairment may not be the same in all three (Bishop 2008; Bishop & Baird 2001). Nonetheless, at least some children with SLI manifest language problems which are common in autism, and *vice versa* (Bishop 2003). Moreover, several genetic overlaps between SLI, autism and ADHD have been observed, the most prominent of which is the implication of the *CNTNAP2* gene in all three disorders (Alarcon *et al.* 2008; Elia *et al.* 2010; Vernes *et al.* 2008). Bishop proposed a model which can account for the genetic overlaps and the phenotypic similarities and dissimilarities between these disorders (Bishop 2010). This model incorporates gene–gene interactions and does not rely solely on additive effects. In line with this model, it is plausible that the causal variants underlying the detected associations play a role in genetic pathways that are shared between the three disorders. While the aforementioned genetic studies mention parent-of-origin effects only in the context of autism, there have been reports of parent-of-origin effects (namely, paternal over-transmission of risk alleles) in studies of ADHD candidate genes (Hawi *et al.* 2010, 2005). However, other studies failed to confirm overall parent-of-origin effects in ADHD (Anney *et al.* 2008; Kim *et al.* 2007). A recent GWAS of parent-of-origin effects in ADHD identified several loci that showed such effects, but none were on chromosome 5 (Wang *et al.* 2012).

Further support for parent-of-origin effects in SLI and other neurodevelopmental disorders will require additional replication cohorts of individuals with neurodevelopmental disorders for whom parental genetic data are also available; we replicated the association on chromosome 5, but in order to attempt to replicate the association on chromosome 14, paternal genotypes need to be available. Moreover, it should be noted that the SLIC cohort is not a large one, which affects the power of the analyses to detect true associations. This is of particular importance in the context of the child trend analysis discussed in this article.

In conclusion, this article presents novel evidence for parent-of-origin effects in SLI. Furthermore, the loci identified in this study overlapped with regions in other neurodevelopmental disorders, supporting the notion of shared and imprinted genetic pathways across several neurodevelopmental disorders.

## Supporting Information

Appendix S1 Annotated summary for top associations.
